# Polydipsia and autistic traits in patients with schizophrenia spectrum disorders

**DOI:** 10.3389/fpsyt.2023.1205138

**Published:** 2023-07-06

**Authors:** Hiroshi Komatsu, Takashi Ono, Yuji Onouchi, Goh Onoguchi, Yoshinori Maita, Yusuke Ishida, Takahiro Maki, Akiko Oba, Hiroaki Tomita, Yoshihisa Kakuto

**Affiliations:** ^1^Department of Psychiatry, Tohoku University Hospital, Sendai, Japan; ^2^Department of Psychiatry, Graduate School of Medicine, Tohoku University, Sendai, Japan; ^3^Miyagi Psychiatric Center, Natori, Japan; ^4^Department of Community Psychiatry, Graduate School of Medicine, Tohoku University, Sendai, Japan

**Keywords:** schizophrenia spectrum disorder, polydipsia, autistic traits, male gender, autism-spectrum quotient

## Abstract

**Introduction:**

Polydipsia, prevalent in 6%–20% of patients with schizophrenia, results in seclusion and prolonged hospitalization. It is also observed in autistic individuals, with previous studies reporting that autism accounted for 20% of all hospitalized patients with polydipsia. The current study investigated the association between polydipsia and autistic traits in patients with schizophrenia spectrum disorders (SSDs) based on the hypothesis that higher autistic traits would be observed in schizophrenic patients with polydipsia.

**Methods:**

In the first study (study A), the autism-spectrum quotient [(AQ); Japanese version] scores of long-stay inpatients with and without polydipsia were compared. Furthermore, the association between polydipsia and autistic traits was also examined in short-stay inpatients and outpatients with SSDs (study B).

**Results:**

Study A showed that patients with polydipsia scored significantly higher on the three AQ subscales (attention switching; communication; and imagination) compared to those without. Study B also showed that patients with polydipsia had significantly higher AQ scores overall and for several subscales compared to those without polydipsia. Binary logistic regression analysis of the combined sample showed that male gender and higher autistic traits were significant predictors of polydipsia.

**Discussion:**

The study highlights the importance of focusing on such traits to understand the pathogenesis of polydipsia in SSD patients.

## 1. Introduction

Schizophrenia is a chronic disease that presents with a variety of symptoms such as cognitive dysfunction. The prevalence of schizophrenia is approximately 1%, with 6%–20% of patients also exhibiting polydipsia (1, 2). Furthermore, 14%–31% of patients with polydipsia may experience hyponatremia (3–6). Polydipsia and water intoxication can lead to seclusion of the patient and prolonged hospitalization. Hawken et al. (7), found that polydipsia also affected mortality in schizophrenic patients, with their life expectancy being approximately 13% shorter than schizophrenic patients without polydipsia. However, the pathogenesis of polydipsia in patients with psychiatric disorders remains unclear. Previous studies identified schizophrenia, prolonged hospitalization, and smoking as risk factors for polydipsia, with Caucasian races and male gender also exhibiting a higher risk of developing water intoxication (6). Schizophrenic patients with polydipsia or water intoxication exhibit a variety of negative symptoms and disorganization (8). The association between polydipsia and antipsychotic drugs remains unclear (6, 9). Kirino et al. (9) reported a systematic review of clinical studies and case reports regarding antipsychotics in polydipsia patients. They reported that 75 (90.3%) of 83 cases who had received antipsychotic medications before developing polydipsia were prescribed first-generation antipsychotics, particularly haloperidol, which was the most prescribed in 24 cases (28.9%). In addition, 36 (90%) of the 40 patients whose polydipsia improved with antipsychotic treatment were prescribed second-generation antipsychotics, most frequently clozapine, which was prescribed in 14 (35%) cases (9). Although there are few controlled clinical studies and the evidence is limited, it is suggested that typical antipsychotics with high affinity for D_2_ receptors may increase the risk of polydipsia (9, 10).

Polydipsia is also observed in patients with other neurodevelopmental disorders or intellectual disabilities, (5) and a previous study investigating patients hospitalized for polydipsia found that approximately 20% of them were autistic (1).

A meta-analysis showed that autistic traits, measured using the autism-spectrum quotient (AQ), were more common in patients with schizophrenia spectrum disorders (SSDs) compared to healthy controls (11). Autistic traits in patients with schizophrenia or psychotic disorders have been shown to be correlated with lower functional outcomes and subjective recovery (12–15).

Therefore, the current study compared autistic traits among schizophrenic patients with and without polydipsia who were hospitalized for prolonged periods of time (study A). Additionally, autistic characteristics were also examined in patients with SSDs with and without polydipsia, who were not long-stay inpatients (study B). The hypothesis being tested was that a higher frequency of autistic traits would be associated with polydipsia in patients with schizophrenia.

## 2. Materials and methods

### 2.1. Participants

Between December 2017 and January 2023, 115 patients were hospitalized for more than 1 year and less than 5 years, of which 30 patients consented to participating in research. Study A included 25 patients with schizophrenia who had been hospitalized for prolonged periods of time (average length of hospital stay: 23.04 ± 13.75 months). Two psychiatrists diagnosed schizophrenia using the Diagnostic and Statistical Manual of Mental Disorders, Fifth Edition (DSM-5), and patients with drug dependence, and those under 20 years of age were excluded from the study.

Study B investigated the association between polydipsia and autistic traits in 110 patients [53 short-hospital-stay patients admitted to the acute care ward (average length of hospital stay: 53.0 ± 66.0 days) and 57 outpatients at the Miyagi Psychiatric Center] with SSD recruited between March and November 2019 (15). Psychological assessment data was collected from 105 patients using a method described previously, as three patients withdrew consent and two did not complete the questionnaires (14). Patients participating in study A and those with comorbid substance use disorders were excluded from study B.

Written informed consent was obtained from all participants and the study was carried out in accordance with the Declaration of Helsinki (1991). The study was approved by the Ethics Committee of the Miyagi Psychiatric Center (MPC-20190320).

### 2.2. Assessment of psychiatric symptoms

In study A, the severity of psychiatric symptoms was assessed by a psychiatrist using the brief psychiatric rating scale (BPRS-18) wherein 18 items were assigned scores using an eight-point scale (0: not assessed; 1: not at all; 7: extremely severe). Total scores ranged from 0 to 126, with higher scores indicating greater symptom severity (16, 17).

### 2.3. Assessment of premorbid estimated intelligence quotient

In study A, the premorbid estimated intelligence quotient (IQ) was assessed using the Japanese adult reading test (JART) developed by Matsuoka et al. (18). It represents the Japanese version of the National adult reading test [NART; developed by Nelson and Willison (19)] and the validity of the shorter version of the JART (JART-25) for estimating the premorbid IQ of patients with schizophrenia has been previously confirmed by Uetsuki et al. (20)

### 2.4. Assessment of cognitive function

In study A, cognitive functions were assessed using the Japanese version of the brief assessment of cognition in schizophrenia (BACS-J) which is a comprehensive measurement of cognitive impairment in schizophrenic patients (21, 22). It consists of six domains that assess verbal memory; working memory; motor function; speed and attention; verbal fluency; and executive function. Each domain is assigned a *z*-score, and the composite score is calculated as the sum of all *z*-scores.

### 2.5. Assessment of autistic traits

In both study A and B, autistic traits were assessed using the Japanese version of the autism-spectrum quotient (AQ-J) developed by Baron-Cohen et al. (23). It is a 50-item self-reported scale that includes five subscales (social skills, attention switching, attention to detail, communication, and imagination), and the reliability and validity of the Japanese version, developed by Wakabayashi et al. (24), has been confirmed previously. The maximum score is 50 points (cut-off: 33 points), and higher scores indicate a stronger tendency toward autistic spectrum disorder.

### 2.6. Dose of psychotropic drug

In both study A and B, antipsychotic drugs were converted to chlorpromazine equivalent doses; (25–28) anxiolytics and hypnotics to diazepam equivalent doses (25); and anti-parkinsonian drugs to biperiden equivalent doses (25). Furthermore, chlorpromazine equivalents were calculated separately for first- and second-generation antipsychotic drugs.

### 2.7. Definition of polydipsia

Polydipsia was diagnosed based on the presence of the term in medical records or staff reports; normalized diurnal weight gain (NDWG) >4%; or evidence of drinking more than 3 liters of water per day. Normalized diurnal weight gain is the percentage of daily weight gain, and NDWG >4% is associated with a higher risk of water intoxication (6).

### 2.8. Statistical analysis

Fisher’s exact test was used to compare sex, smoking, diagnosis, inpatient or outpatient status, and diabetes mellitus. among patients with and without polydipsia. Age; age of disease onset; number of hospitalizations; antipsychotic drug dosage; BPRS scores; JART-25 premorbid estimated IQ; BACS *z*-scores; and AQ scores were compared between the two groups using the Mann–Whitney *U* test or unpaired *t*-test. Finally, a binary logistic regression analysis was used to examine the association between polydipsia and autistic traits in a combined sample of patients from both studies, and gender, age, age of disease onset, and the number of hospitalizations were included as covariates. Statistical analysis was performed using IBM SPSS Statistics, version 24 (IBM Japan, Tokyo, Japan).

## 3. Results

In study A, 28% (7/25) of long-hospital-stay patients were diagnosed with polydipsia. Three patients without polydipsia were diagnosed with comorbid autism spectrum disorders or intellectual disability. All patients with polydipsia had no diagnosis of diabetes (Table 1). No significant differences in gender; age; number of hospitalizations; smoking status; BPRS total and subscale scores; JART-25 premorbid estimated IQ; and *z*-scores for each cognitive domain of BACS were observed between the two groups (Table 1). On the other hand, patients with polydipsia had taken no first-generation antipsychotics (FGA) and had significantly lower doses of prescribed FGA. Furthermore, they also exhibited significantly higher scores on three subscales (attention switching, communication skills, and imagination) of the AQ-J compared to patients without polydipsia (Table 1).

**Table 1:** Demographic and clinical characteristics of schizophrenic patients with and without polydipsia in study A (including long-stay inpatients).

	Patients with polydipsia (*n* = 7)	Patients without polydipsia (*n* = 18)	*p*-value
Sex (male/female)^a^	4/3	10/8	1.00
Age (mean ± SD, years)^b^	38.57 ± 12.23	44.56 ± 16.31	0.326
*Psychiatric comorbidity* ^a^			
Autism spectrum disorder	0	2	0.51
Intellectual disability	0	1	0.72
Age of onset (mean ± SD)^b^	20.00 ± 7.19	23.47 ± 10.86	0.383
Period of hospitalization (mean ± SD, month)^b^	22.14 ± 15.24	23.39 ± 13.59	0.574
Number of hospitalizations (mean ± SD)^b^	4.43 ± 2.149	4.88 ± 5.92	0.455
Smoking (yes/no)^a^	0/7	3/15	0.534
Diabetes mellitus^a^	0	0	–
*Psychotropic medication* ^c^			
Dose of antipsychotics (SGA + FGA) (CP equivalent, mean ± SD, mg/day)	981.43 ± 401.51	858.56 ± 472.443	0.55
Dose of SGA (CP equivalent, mean ± SD, mg/day)	960.71 ± 347.57	674.94 ± 497.21	0.179
Dose of FGA (CP equivalent, mean ± SD, mg/day)	0	211.94 ± 360.52	**0.023**
Dose of anxiolytics and hypnotics (diazepam equivalent, mean ± SD, mg/day)	9.52 ± 7.74	11.22 ± 12.66	0.745
Dose of antiparkinsonian drugs (biperiden equivalent, mean ± SD, mg/day)	0.57 ± 1.51	1.22 ± 2.21	0.484
*BPRS* ^b^			
Total score (mean ± SD)	46.43 ± 9.43	47.83 ± 17.31	0.929
*Subscale*			
Positive symptoms (mean ± SD)	13.14 ± 2.97	13.33 ± 6.44	0.929
Negative symptoms (mean ± SD)	8.43 ± 3.82	9.50 ± 4.69	0.615
Hostile-uncooperativeness (mean ± SD)	9.00 ± 2.24	9.11 ± 4.58	0.657
Depression-anxiety (mean ± SD)	6.86 ± 3.19	6.44 ± 3.11	0.790
*JART-25* ^b^			
Premorbid estimated FIQ (mean ± SD)	86.86 ± 4.38	93.35 ± 12.16	0.318
Premorbid estimated VIQ (mean ± SD)	85.29 ± 5.09	93.06 ± 13.82	0.288
Premorbid estimated FIQ (mean ± SD)	89.57 ± 3.55	94.76 ± 9.21	0.260
*BACS (z-score)* ^b^			
Verbal memory (mean ± SD)	−3.30 ± 1.38	−3.09 ± 1.59	0.852
Working memory (mean ± SD)	−2.24 ± 1.18	−1.70 ± 1.92	0.249
Motor function (mean ± SD)	−2.51 ± 1.68	−3.18 ± 1.64	0.590
Speed and attention (mean ± SD)	−2.87 ± 1.87	−3.62 ± 2.26	0.222
Verbal fluency (mean ± SD)	−2.24 ± 1.12	−1.90 ± 1.42	0.624
Executive function (mean ± SD)	−0.756 ± 1.73	−2.75 ± 2.89	0.179
Composite score (mean ± SD)	−2.10 ± 0.89	−2.54 ± 1.50	0.547
*AQ* ^b^			
Total score	29.43 ± 4.86	23.67 ± 6.29	0.106
*Subscale score*			
Social skills	6.43 ± 2.57	4.50 ± 2.50	0.135
Attention switching	6.14 ± 2.04	4.35 ± 1.58	**0.028**
Attention to detail	3.71 ± 2.14	5.40 ± 2.20	0.106
Communication skills	6.71 ± 1.89	4.41 ± 2.27	**0.047**
Imagination	6.43 ± 1.27	4.29 ± 1.53	**0.004**

SGA, second-generation antipsychotics; FGA, first-generation antipsychotics; BPRS, brief psychiatric rating scale; JART-25, Japanese adult reading test, the abridged version; FIQ, full-scale intelligence quotient; VIQ, verbal intelligence quotient; PIQ, performance intelligence quotient; BACS, brief assessment of cognition in schizophrenia; AQ, autism-spectrum quotient.

a Fisher’s exact test *p*-value for comparison of sex, diagnosis, and smoking status between the two groups.

b Mann–Whitney *U* test *p*-value for comparison of age, age of onset, period of hospitalization, number of hospitalizations, BPRS, JART-25, BACS, or AQ between the two groups.

c Unpaired *t*-test *p*-value for comparison of the use of psychotropic medications between the two groups.

Bold values indicate statistical significance (*p* < 0.05).

In study B, 12.4% of patients [outpatients (*N* = 55) and short-stay inpatients (*N* = 50)] were diagnosed with polydipsia (outpatients: 7.8%; inpatients: 22%; Table 2; Supplementary Table S1). One patient with polydipsia was diagnosed with a comorbid autism spectrum disorder. One of the 13 patients with polydipsia had been diagnosed with diabetes mellitus (Table 2). A significantly higher proportion of patients with polydipsia was male and smoked compared to those without polydipsia (Table 2). Furthermore, these patients also exhibited onset of disease at a younger age and were hospitalized more frequently than patients without polydipsia (Table 2). Patients with polydipsia had significantly higher diazepam equivalents of anxiolytics and hypnotics and biperiden equivalents of antiparkinsonian drugs than patients without polydipsia (Table 2). In both studies, patients with polydipsia exhibited significantly higher AQ scores overall and for two subscales (attention switching and communication skills) in particular compared to patients without polydipsia (Table 2).

**Table 2:** Demographic characteristics and autistic traits among patients with and without polydipsia in study B.

	Patients with polydipsia (*n* = 13)	Patients without polydipsia (*n* = 92)	*p*-value
Sex (male/female)^a^	11/2	37/55	**0.005**
Age (mean ± SD)^b^	41.23 ± 12.99	47.80 ± 13.26	0.100
Diagnosis^a^			0.745
Schizophrenia	13	88	
Schizoaffective disorder	0	2	
Delusional disorder	0	2	
*Psychiatric comorbidity* ^a^			
Autism spectrum disorder	1	0	0.124
Intellectual disability	0	1	0.876
Inpatient/outpatient^a^	9/4	41/51	0.138
Age of onset (mean ± SD)^b^	22.23 ± 7.57	29.72 ± 11.63	**0.016**
Number of hospitalizations (mean ± SD)^b^	7.33 ± 5.43	3.57 ± 4.90	**0.016**
Smoking (yes/no)^a^	10/3	33/59	**0.007**
Marital status (married/unmarried/divorce)^a^	0/12/1	20/59/13	0.104
Diabetes mellitus^a^	1	7	0.666
*Psychotropic medication* ^c^			
Dose of antipsychotics (SGA + FGA) (CP equivalent, mean ± SD, mg/day)	744.19 ± 327.00	570.54 ± 346.44	0.092
Dose of SGA (CP equivalent, mean ± SD, mg/day)	648.50 ± 319.54	548.89 ± 333.82	0.314
Dose of FGA (CP equivalent, mean ± SD, mg/day)	95.69 ± 156.60	21.71 ± 82.96	0.119
Dose of anxiolytics and hypnotics (diazepam equivalent, mean ± SD, mg/day)	11.06 ± 7.66	6.04 ± 7.44	**0.025**
Dose of antiparkinsonian drugs (biperiden equivalent, mean ± SD, mg/day)	1.77 ± 1.69	0.82 ± 1.55	**0.042**
*AQ* ^b^			
Total score	28.54 ± 6.20	23.20 ± 6.19	**0.005**
*Subscale score*			
Social skills	5.62 ± 2.26	4.73 ± 2.38	0.218
Attention switching	6.23 ± 1.48	5.13 ± 1.87	**0.039**
Attention to detail	5.54 ± 1.94	5.24 ± 2.17	0.575
Communication skills	5.69 ± 2.43	3.85 ± 2.19	**0.013**
Imagination	5.46 ± 1.61	4.25 ± 2.05	0.053

CP, chlorpromazine; SGA, second-generation antipsychotics; FGA, first-generation antipsychotics; AQ, autism-spectrum quotient.

a Fisher’s exact test *p*-value for comparison of sex, diagnosis, inpatients/outpatients, smoking status, and marital status between the two groups.

b Mann–Whitney *U* test *p*-value for comparison of age, age of onset, number of hospitalizations, and AQ between the two groups.

c Unpaired *t*-test *p*-value for comparison of the use of psychotropic medication between the two groups.

Bold values indicate statistical significance (*p* < 0.05).

In the combined sample from both studies, a higher proportion of patients with polydipsia were males, exhibited onset of disease at a younger age, and were hospitalized more frequently compared to those without polydipsia (Table 3). No significant differences in smoking status and the use of psychotropic medications were observed between the two groups (Table 3). AQ total and subscale scores (excluding the attention to detail subscale) were significantly higher in polydipsia patients compared to those without polydipsia (Table 3). The binary logistic regression analysis adjusted for sex, age, age of disease onset, and the number of hospitalizations showed that the total AQ score was an independent predictor of polydipsia (Table 4).

**Table 3:** Demographics characteristics and autistic traits among SSD patients with and without polydipsia in the combined sample (studies A and B).

	Patients with polydipsia (*n* = 20)	Patients without polydipsia (*n* = 110)	*p*-value
Sex (male/female)^a^	15/5	47/63	**0.013**
Age (mean ± SD)^b^	40.30 12.469	47.27 13.772	**0.041**
Diagnosis^a^			0.687
Schizophrenia	20	106	
Schizoaffective disorder	0	2	
Delusional disorder	0	2	
*Psychiatric comorbidity* ^a^			
Autism spectrum disorder	1	2	0.398
Intellectual disability	0	2	0.715
Inpatient/outpatient^a^	9/11	41/69	0.619
Age of onset (mean ± SD)^b^	21.45 ± 7.33	28.74 ± 11.69	**0.003**
Number of hospitalizations (mean ± SD)^b^	6.26 ± 4.65	3.78 ± 5.06	**0.005**
Smoking (yes/no)^a^	10/10	36/74	0.202
Diabetes mellitus^a^	1	7	0.644
*Psychotropic medication* ^c^			
Dose of antipsychotics (SGA + FGA) (CP equivalent, mean ± SD, mg/day)	676.25 ± 414.34	601.14 ± 386.58	0.431
Dose of SGA (CP equivalent, mean ± SD, mg/day)	641.25 ± 369.32	552.94 ± 373.58	0.332
Dose of FGA (CP equivalent, mean ± SD, mg/day)	27.75 ± 105.90	52.88 ± 173.61	0.533
Dose of anxiolytics and hypnotics (diazepam equivalent, mean ± SD, mg/day)	10.56 ± 7.51	8.81 ± 9.34	0.062
Dose of antiparkinsonian drugs (biperiden equivalent, mean ± SD, mg/day)	0.55 ± 1.15	1.15 ± 1.93	0.428
*AQ* ^b^			
Total score	28.85 ± 5.65	23.26 ± 6.17	**<0.001**
*Subscale score*			
Social skills	5.90 ± 2.34	4.69 ± 2.39	**0.047**
Attention switching	6.20 ± 1.64	5.01 ± 1.84	**0.005**
Attention to detail	4.90 ± 2.15	5.26 ± 2.17	0.478
Communication skills	6.05 ± 2.26	3.94 ± 2.20	**<0.001**
Imagination	5.80 ± 1.54	4.26 ± 1.97	**0.002**

SGA, second-generation antipsychotics; FGA, first-generation antipsychotics; AQ, autism-spectrum quotient.

a Fisher’s exact test *p*-value for comparison of sex, diagnosis, inpatients/outpatients, and smoking status between the two groups.

b Mann–Whitney *U* test *p*-value for comparison of age, age of onset, number of hospitalizations, and AQ between the two groups.

c Unpaired *t*-test *p*-value for comparison of the use of psychotropic medication between the two groups.

Bold values indicate statistical significance (*p* < 0.05).

**Table 4:** Binary logistic regression analysis in the combined sample.

Predictor variables	*β*	SE	Wald	*p*-value	Exp (*B*)
Sex	−2.022	0.735	7.577	**0.006**	0.132
Age	−0.049	0.03	2.744	0.098	0.952
Age of onset	−0.005	0.046	0.013	0.911	0.995
Number of hospitalizations	0.101	0.052	3.717	0.054	1.106
AQ total score	0.172	0.055	9.626	**0.002**	1.187

Bold values indicate statistical significance (*p* < 0.05).

## 4. Discussion

The findings of the current studies showed that patients with polydipsia exhibited higher autistic traits than patients without polydipsia. Moreover, autistic traits (measured by AQ) and male gender were associated with polydipsia in patients with SSDs. These findings highlight the importance of focusing on autistic symptoms when trying to understand the pathophysiology of polydipsia in patients with SSDs.

The pathogenesis of polydipsia remains unclear to date. Leon et al. (6), found that prolonged hospitalization and smoking were associated with polydipsia, and this was supported by the findings of study B where polydipsia patients exhibited significantly higher rates of smoking. Moreover, the frequency of polydipsia in long- and short-hospital stay inpatients were 24% and 22%, respectively, and this was also generally consistent with the proportion of inpatients diagnosed with polydipsia using staff reports in the study by Leon et al. (6). In the current study, 7.8% of outpatients exhibited polydipsia and this was lower than that previously reported by Iftene et al. (29). However, they diagnosed polydipsia using the specific gravity of urine (SPGU) and the structured interview method proposed by Nilsson et al. (30), while the current study carried out diagnosis using the medical records of outpatients only. Therefore, there is a possibility that the prevalence of polydipsia in outpatients may have been underestimated in the current study.

The age of onset of disease was also found to be associated with polydipsia in the current study. Poirier et al. (31), previously investigated the clinical characteristics of 12 schizophrenic patients with polydipsia and water intoxication and observed a higher frequency of severe psychotic symptoms, earlier onset of disease, and prior history of alcohol misuse. In study A, no association between polydipsia and psychiatric symptoms, cognitive deficits, or premorbid estimated IQ was observed. However, this could likely be attributed to insufficient statistical power due to the limited number of cases included in the study, and further validation of this finding is warranted.

Typical antipsychotic drugs with high D_2_ receptor blockade have been shown to be associated with a higher risk of polydipsia (9, 10, 32). A potential explanation is that the blockade of postsynaptic dopamine receptors by antipsychotic drugs causes hypersensitivity to the receptors, resulting in increased presynaptic dopamine release which, in turn, increases dopamine activity in the hypothalamus (thirst center) leading to polydipsia (33). Conversely, atypical antipsychotics with low affinity for D_2_ receptors, such as clozapine, appear to be effective for polydipsia (9, 34, 35). First-generation antipsychotic drugs were not prescribed in patients with polydipsia in study A, while no significant differences in chlorpromazine equivalents of first-generation antipsychotic drugs were observed between patients with and without polydipsia in study B. Although the cross-sectional design of these studies prevents establishment of a causal relationship, it is possible that the presence of polydipsia or water intoxication led to increased prescription of second-generation antipsychotic drugs. Antipsychotics, especially second-generation antipsychotics, have been reported to increase the risk of diabetes (36). One patient with polydipsia in study B had been diagnosed with diabetes mellitus. Although hyperglycemia is known to cause thirst, we confirmed that the patient’s blood glucose was under control. Patients with polydipsia were prescribed higher doses of hypnotics, anxiolytics, and antiparkinsonian medications, and these drugs, along with typical antipsychotics, may be considered to increase the risk of polydipsia. The anticholinergic effects of anxiolytics, hypnotics, and antiparkinsonian drugs may have partially contributed to polydipsia, suggesting that a reduction in dosage could be a potential preventive measure.

The findings of the current studies showed that schizophrenic patients with polydipsia exhibited autistic traits more frequently than those without polydipsia, and this was in agreement with a previous brain imaging study that showed a reduction in volume in the left insula in schizophrenic patients with polydipsia compared to those without (37). The insula plays an important role in self-awareness, theory of mind, and the distinction between self and non-self, which underlies appropriate interpersonal relationships and the interpretation and understanding of self and reality (38). Impairment of these functions constitute autistic traits in patients with schizophrenia. Previous studies have reported reduced insula volume, dysfunction, and impaired functional connectivity with other areas in patients with autism spectrum disorders (38–40), suggesting that polydipsia and autistic traits share a common brain neural basis in schizophrenic patients. Previous genetic studies have identified shared genetic variants in both schizophrenia and autism spectrum disorders (e.g., neurexin family gene, *CNTNP2*, *SHANK2* gene) (41). Interestingly, shared genetic variants include the OXTR (oxytocin receptor)/OXT (oxytocin) gene. De Berardis et al. (42), found that plasma oxytocin levels were lower in schizophrenic patients with polydipsia and hyponatremia compared to those without. Oxytocin, a neuropeptide that regulates social activity and cognition, is a potential treatment for autism spectrum disorders (43). This suggests that the association between autistic traits and polydipsia could also be attributed to oxytocin dysfunction. The effects of other genes such as the neurexin family gene, *CNTNAP2*, and *SHANK2* gene on polydipsia remained to be clarified. In the future, monitoring the amount of water consumed by animals with the related mutations and analysis of the related gene polymorphisms in polydipsia may be useful in elucidating the biological mechanisms of polydipsia.

To the best of our knowledge, the current study is the first to identify an association between polydipsia and autistic traits in patients with SSDs. Elucidation of the shared biological mechanisms underlying this association may lead to a better understanding of the pathogenesis of polydipsia and the development of novel therapeutic strategies.

The current study had several limitations. Firstly, it included a relatively small sample size that was restricted to a single medical institution, and future studies should include a larger number of patients from multiple centers to improve the generalizability of the findings. Secondly, autistic traits were assessed using self-reported scales instead of interviewer rating scales such as the parent-interview autism spectrum disorders rating scales, text revision (PARS-TR) or the autism diagnostic observation schedule, second edition (ADOS-2). Finally, SPGU was not assessed and this may have led to underestimation of the prevalence of polydipsia.

In conclusion, the current study is the first to show an association between autistic traits and polydipsia in patients with SSDs, highlighting the importance of focusing on such traits when trying to understand the pathogenesis of polydipsia in patients with SSDs. Further studies using larger sample sizes are necessary to confirm these findings.

## Data availability statement

The raw data supporting the conclusions of this article will be made available by the authors, without undue reservation.

## Ethics statement

The studies involving human participants were reviewed and approved by The Ethics Committee of the Miyagi Psychiatric Center (MPC-20190320). The patients/participants provided their written informed consent to participate in this study.

## Author contributions

HK contributed to the study design, data acquisition, analysis, and interpretation, and drafting of the manuscript. YO, YM, YI, TM, and AO were involved in data acquisition. TO, YK, GO, and HT reviewed the intellectual content of the manuscript. All authors contributed to the article and approved the submitted version.

## Funding

This work was supported by JSPS KAKENHI grant number 22K07551.

## Conflict of interest

The authors declare that the research was conducted in the absence of any commercial or financial relationships that could be construed as a potential conflict of interest.

## Publisher’s note

All claims expressed in this article are solely those of the authors and do not necessarily represent those of their affiliated organizations, or those of the publisher, the editors and the reviewers. Any product that may be evaluated in this article, or claim that may be made by its manufacturer, is not guaranteed or endorsed by the publisher.
