# Supplementary material for: Polydipsia and autistic traits in patients with schizophrenia spectrum disorders
Source: Front Psychiatry. 2023 Jul 6;14:1205138. doi: 10.3389/fpsyt.2023.1205138 (PMC10359144; doi:10.3389/fpsyt.2023.1205138)
Supplement: Supplementary file 1 [file Table_1.DOCX]

**Supplementary Table 1: Demographic characteristics of patients in Study B**

|  | **Total sample (N = 105)** | **Outpatients (N = 55, 52.3%)** | **Short-stay inpatients (N = 50, 47.7%)** | **p value** |
| --- | --- | --- | --- | --- |
| Sex (male/female) ^a^ | 48/57 | 22/33 | 26/24 | 0.244 |
| Age ^b^ | 47.0 ± 13.3 | 47.8 ± 14.2 | 46.1 ± 12.4 | 0.529 |
| Diagnosis ^a^ |  |  |  |  |
| Schizophrenia | 101 | 53 | 48 | 1 |
| Schizoaffective disorder | 2 | 0 | 2 |  |
| Delusional disorder | 2 | 2 | 0 |  |
| Age of onset ^b^ | 28.8 ± 11.5 | 30.2 ± 11.7 | 27.3 ± 11.1 | 0.156 |
| Duration of hospitalization (days) | 53.0 ± 66.0 |  | 53.0 ± 66.0 |  |
| Number of hospitalizations ^c^ | 4.0 ± 5.1 | 3.5 ± 4.7 | 4.7 ± 5.5 | 0.528 |
| Polydipsia (+)/Polydipsia (-) ^a^ | 13/92 | 4/51 | 9/41 | 0.138 |

Notes:

^a^ Fisher's exact test p-value for comparison of sex, diagnosis, and polydipsia between the two groups.

^b^ Unpaired t-test p-value for comparison of age and age of disease onset between the two groups.

^c^ Mann-Whitney U test p-value for comparison of the number of hospitalizations between the two groups.
